# Validation of ocular trauma score as a prognosticator of visual outcomes in open and closed-globe injuries at Kaduna, Nigeria

**DOI:** 10.1186/s12886-026-05509-x

**Published:** 2026-11-04

**Authors:** Thelma Imaobong Ndife, Ahmed Saidu, Muhammad Nasiru, Ahmadu Shehu Mohammed, Joelyn Chiamaka Ndife

**Affiliations:** 1 Department of Ophthalmology, University of Uyo Teaching Hospital, Uyo, Akwa Ibom Nigeria; 2 Headquarters Nigerian Air Force Medical Services, Abuja, Nigeria; 3 Usman Danfodio University Teaching Hospital, Sokoto, Nigeria; 4 Nigerian Navy Reference Hospital, Calabar, Nigeria

**Keywords:** Ocular trauma score, Visual acuity, Comparative, Open-globe injury, Closed-globe injury

## Abstract

**Background:**

Prognostic tools, including the ocular trauma score, are critical for clinicians in guiding management decisions and counseling patients about visual prognosis. This concern is especially relevant in developing communities with a high burden of visual impairment and blindness due to ocular trauma. This study compared the predictive value of the ocular trauma score (OTS) with actual visual outcomes following closed and open-globe injuries.

**Method:**

A prospec​tive comparative analysis of Kuhn et al.’s ocular trauma score-predicted visual acuity and final visual outcome was conducted. Patients with mechanical ocular trauma managed at a tertiary care facility in Nigeria were enrolled. Ocular injuries were classified according to the Birmingham Eye Trauma Terminology System (BETTS). The primary outcome was final visual acuity at 3 months post-presentation. The association between presentin​g and final visual acuity and the correlation between the ocular trauma score predicted and final visual acuity were analyzed.

**Result:**

Seventy-nine patients participated, with 34.2% sustaining open-globe injuries and 65.8% closed-globe injuries; the male-to-female ratio was 2.8:1. The mean age was 21.16 years (SD ± 12.63). Approximately 31.6% presented within 24 h of injury. Injury types included contusions (59.5%), penetrating injuries (16.5%), ruptured globe (13.9%), lamellar lacerations (6.3%), and intraocular foreign bodies (3.8%). Zonal distribution was 30.4% in Zone 1, 48.1% in Zone II, and 21.5% in Zone III. Medical management was provided to 49.3% of patients, while 38% un​derwent surgical intervention. Presenting visual acuity was hand motion or worse in 48.7% of patients and 6/12 or better in 23.1%. Final visual acuity was 6/12 or better in 58% of patients. The presenting visual acuity showed association (*p* < 0.001) and correlation (Spearman’s *r* = 0.53, p = < 0.001, 95% CI [0.32, 0.69]) with the final visual acuity. The ocular trauma score predicted and the final visual outcomes were not statistically different (*p* = 0.835) but showed positive correlation (Spearman’s *r* = 0.60, p = < 0.001, 95% CI [0.42, 0.74]).

**Conclusion:**

The visual acuity trends predicted by the ocular trauma score were consistent with the outcomes observed in this study. The ocular trauma score is a valuable predictor of final visual outcome three months after open- and closed-globe injuries.

**Clinical trial number:**

Not applicable.

**Supplementary Information:**

The online version contains supplementary material available at https://doi.org/10.1186/s12886-026-05509-x.

## Introduction

Ocular trauma is one of the main causes of monocular visual impairment and blindness [1, 2]. The World Health Organization (WHO) estimates that 750,000 individuals with eye injuries are hospitalized globally each year [3]. Despite comprising only 0.1% of the total body surface and 0.27% of the anterior body surface, the function of the eye is disproportionately highly significant [4]. Ocular injuries frequently result in discomfort, pain, vision loss, increased health care costs, and lost productivity [5]. The pattern and etiology of ocular injuries differ across countries and regions and are influenced by age, gender and occupation. Outcomes depend on factors such as the cause and type of injury, the timeliness of medical intervention, and the standard of care delivered. The burden of ocular trauma, with its associated physical, psychological, social, and economic consequences, remains substantial, particularly in developing countries such as Nigeria [6–9].

Visual prognosis following ocular trauma is a primary concern for patients and their families. The Birmingham Eye Trauma Terminology System (BETTS) provides a standardized, straightforward classification of mechanical injuries to the globe [10]. The Ocular Trauma Score (OTS) [11], along with the modified Pediatric Ocular Trauma Score (POTS) [12, 13], estimates potential visual outcomes six months after mechanical trauma. However, prospective studies comparing OTS-predicted visual outcomes with actual outcomes are limited, particularly in developing countries. Increasing civil unrest in Northern Nigeria may have influenced the pattern and outcomes of ocular trauma observed in this region. Accurate prognostication of visual outcomes is essential for timely and influential clinical decision-making. The study intended to compare OTS-predicted visual outcomes with final visual outcomes three months post-intervention for both open-globe and closed-globe injuries. Findings from this comparison are intended to support ophthalmologists in clinical decision-making and inform policymakers addressing the growing burden of ocular trauma in this region.

## Methodology

### Study design

The study was a prospective comparative analysis conducted over 4 months from August 1st to November 30th 2016. The National Eye Centre, Kaduna (NECK) is a tertiary health facility in Northern Nigeria. An average of 4500 new patients passed through the emergency department monthly, of which about 30 were trauma cases [14]. Using the following formula [15]: n = Z2pq/d2, and the prevalence of ocular injuries from a previous study, 4.5% [16]; *n* = 66. For a population < 10,000: nf = n/1+(n/N), where N is the estimated population (the number of trauma cases seen at the emergency department over the last year; 384). At an expected response rate of 70%, the minimum sample size was 80.

### Inclusion / exclusion criteria

All willing new patients with mechanical ocular trauma presenting to the hospital’s emergency department during the study period were eligible. Patients with prior surgery, thermal or chemical injuries or ocular co-morbidities such as diabetic or hypertensive retinopathies and macular pathology which could affect the outcome of ocular trauma and those who were unwilling to participate were excluded.

### Definition of terms

#### The BETTS classification of ocular trauma[10]

In BETTS, the sclera and cornea are considered the eye wall. If an injury is atypical, or ‘mixed’, clinicians can classify based on their best judgment or on the injury with the worst prognosis. 

Cl​osed-globe injuries are partial-thickness wounds of the eye wall. They are divided into contusion (usually caused by a blunt object) and lamellar laceration (caused by a sharp object). Contusions present with bruising and swelling and may cause some structural changes in the shape of the globe, e.g. angle recession.

Open-globe injuries are full-thickness wounds of the eye wall. The choroid and retina may be intact, damaged or prolapsed. ‘Inside-out’ force by the impact of a blunt object can rupture the eye wall, resulting in tissue herniation at its weakest point. Sharp objects causing ‘outside-in’ mechanisms can result in full-thickness wounds / lacerations at the impact site. A penetrating injury is a single laceration by a sharp object with an entrance and no exit wound, while a perforation has two full-thickness lacerations, i.e., entrance and exit wounds caused by the same object.

#### The ocular trauma score (OTS)[11]

The OTS was used to predict visual outcomes in patients with both closed- and open-globe ocular injuries. OTS scores range from Category 1 (most severe injury and worst prognosis) to Category 5 (least severe injury and best prognosis). Each score was associated with a range of predicted post-injury visual acuities. Patients are assigned scores ranging from 100 to 60 based on their presenting visual acuity. Thereafter, scores are subtracted if the mechanism of injury was due to a ruptured or penetrating injury and if other complications, such as afferent pupillary defect, retinal detachment, and endophthalmitis, are present [11].

### Study procedure

The data collected included socio-demographic (age, gender, occupation) and medical history (time of injury, duration before presentation) of patients. Comprehensive ocular evaluation included visual acuity (VA), intraocular pressure (IOP) measurements, slit lamp examination, and fundoscopy. VA was measured for all adults and older children using the Snellen’s Chart placed at a distance of 6 m from the patient. Kay’s picture chart was used for verbal children under 5 years old. The eyes were examined for relative afferent pupillary defect. Detailed slit lamp examination was carried out to look for the zone of injury, any loss of integrity of the globe wall, hyphema, ruptured lens, and any other abnormality. Dilated fundoscopy, gonioscopy and IOP measurement were carried out on patients with non-penetrating injuries where possible. The IOP was measured using the Tono-pen tonometer in adults and the I-Care rebound tonometer in children. Ocular trauma classification was based on the Birmingham Eye Trauma Terminology System (BETTS) [10]. All patients were assigned scores according to the OTS guidelines. In eyes with a closed globe injury and poor or no view of the fundus, a B-scan ultrasonography was done. Other relevant investigations, such as orbital X-ray and CT scan, were carried out as indicated. Surgical procedures were documented, and patients were contacted using their phone numbers in case of loss to follow-up. All patients were assessed at first presentation, and the best corrected VA at 3 months post presentation was taken as the final visual outcome.

A categorical method [17] of analysis was used to assess the prognostic value of OTS. The final VA was grouped into 5 VA categories identical to the standard OTS categories, with 1–5 corresponding to NLP, LP-HM, CF to < 6/60, 6/60 to < 6/12, and 6/12 or better, respectively. The mean Actual Visual Acuity Rank (AVAR) was calculated for each group by averaging all patients’ VA rankings within the group. For example, a group of 3 patients who had final VA of 6/6, 6/12, and CF would have an AVAR score of (5 + 4 + 3) / 3 = 4. Therefore, theoretically, a group in which every patient had NLP would receive a score of 1, and a group in which all patients had 6/6 vision would receive a score of 5. The OTS-predicted VA (OVAR) was equally ranked. Using the predicted VA percentage distributions by Kuhn et al. [11], OVAR scores were calculated as a weighted average of each VA rank for a given OTS. For example, an OTS of 1 predicts a final VA distribution of 73% NLP, 17% LP-HM, 7% CF to < 6/60, 2% 6/60 to < 6/12, and 1% 6/12 or better; therefore, an injury with OTS 1 would have an OVAR of (0.73 × 1) + (0.17 × 2) + (0.07 × 3) + (0.02 × 4) + (0.01 × 5) = 1.41. Based on this system, OVAR scores closer to 5 were better predictors of outcomes than those closer to 1.

### Data analysis

Data were analyzed using the IBM Statistical Package for Social Science (SPSS) version 22.0 (IBM Corp., Armonk, NY). Categorical variables were presented by frequencies and percentages. Chi square test was used to determine the association between presenting and final VA, and between the final VA and the OTS scores. The strength of the associations was analyzed with Spearman’s correlation coefficient. Comparison of OTS-predicted visual acuity with actual visual outcomes was performed using the Mann-Whitney U test, and p-values ≤ 0.05 were considered statistically significant at the 95% confidence interval level.

## Results

A total of 79 patients participated in the study, including 58 males (73.4%) and 21 females (26.6%). Patient ages ranged from 3 to 60 years, with a mean age of 21.16 years (SD ± 12.63). Table 1, presents the time interval between injury and hospital presentation. Approximately one-third of patients (31.6%) presented within 24 h of injury, and 68.4% selected the hospital as their first point of care. Closed globe injuries (*n* = 52, 65.8%) were more frequent than open globe injuries (*n* = 27, 34.2%). As shown in Tables 1 and 59.5% of injuries were contusions, 16.5% were penetrating injuries (*n* = 13), 13.9% were ruptured globes (*n* = 11), 6.3% were lamellar lacerations (*n* = 5), and 3.8% involved intraocular foreign bodies (*n* = 3).

**Table 1:** Time of presentation to the hospital, type and extent of ocular injuries among patients

Description	​Frequency(*n*)	Percentage (%)
**Time elapsed before presentation**		
Within 24 h	25	31.6
1 – 3 days	14	17.7
3 – 7 days	22	27.8
>7 days	18	22.8
** Total**	**79**	**100**
**Sub type of eye injury**		
Open globe-Rupture	11	13.9
-IOFB (Intraocular foreign body)	3	3.8
-Penetrating	13	16.5
Closed globe-Lamellar laceration	5	6.3
-Contusion	47	59.5
** Total**	**79**	**100**
**Extent of Injury**		Zone Total
Zone 1 - Open-globe	5 (6.3)	24 (30.4)
- Closed- globe	19 (24.1)	
Zone 11 - Open-globe	10 ( 12.7)	38 (48.1)
- Closed- globe	28 (35.4)	
Zone 111 - Open-globe	12 ( 15.2)	16 (21.5)
-Closed- globe	5 (6.3)	
** Total**	**79 (100)**	**79 (100)**

**Fig. 1:**
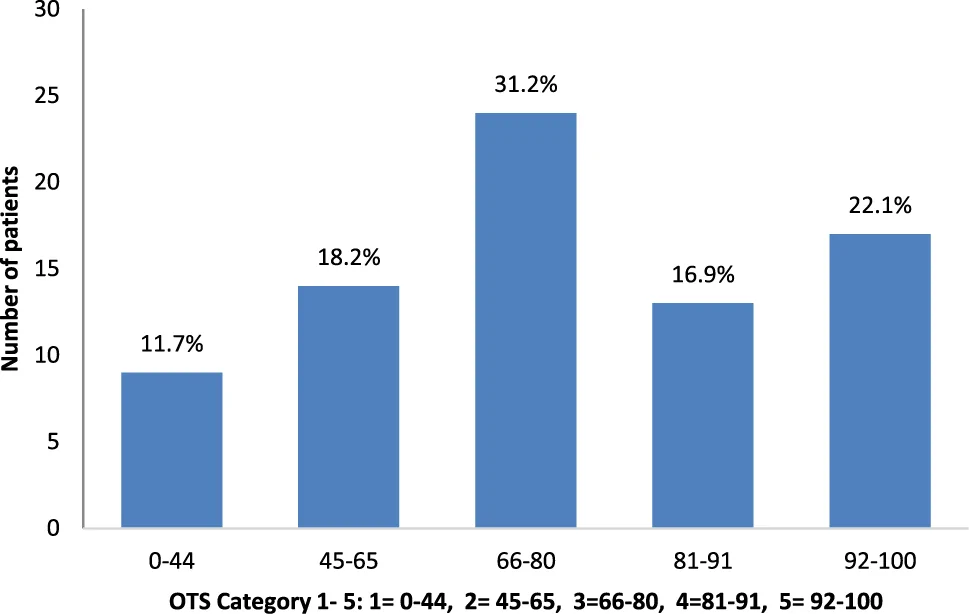
Ocular trauma scores (OTS) of patients at presentation

Among patients with closed-globe injuries, 19 (24.1%) involved zone I, and 28 (35.4%) involved zone II; and 5 (6.3%) involved zone III. For open-globe injuries, 5 (6.3%) were in zone I, 10 (12.7%) in zone II, and 12 (15.2%) in zone III. Figure 1 displays the Ocular Trauma Score (OTS) distribution at presentation. Approximately 31.2% of patients had an OTS raw sum of 66–80 (Category 3) at presentation, while the lowest OTS Category 1 was observed in 11.7% of patients.

**Fig. 2:**
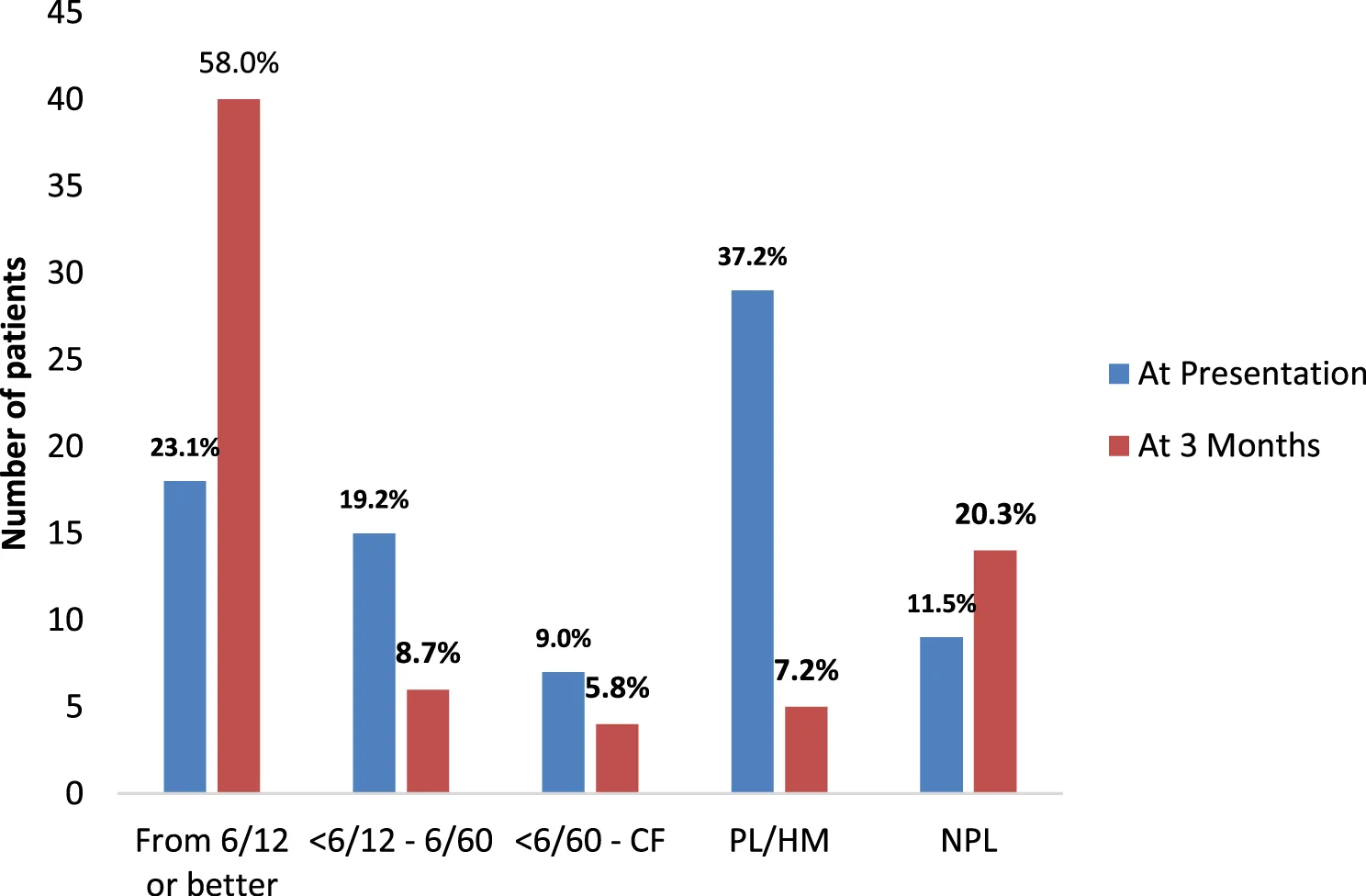
VA at presentation and at 3 months after injury

Medical management was provided to 49.3% of patients, while 38% underwent surgical intervention. A total of 10 patients (12.7%) did not attend the 3-month follow-up. Figure 2 illustrates the presenting visual acuity (VA) and final visual outcomes following ocular trauma. At presentation, 48.7% of patients had VA of ≤ perception of light/hand motion (PL-HM), and 23.1% had VA of ≥ 6/12. After 3 months, 58% of patients achieved VA of ≥ 6/12, while 27.5% had VA of ≤ PL-HM.

Table 2 presents the association between presenting visual acuity and final visual acuity at 3 months. Visual acuity improved significantly at the 3-month follow-up compared to presentation (*p* < 0.001). At 3 months, 40 patients had VA of 6/12 or better, compared to 18 at presentation. The number of patients with VA between 6/60 and < 6/12 decreased from 14 to 6, those with counting fingers (CF) to 6/60 decreased from 7 to 4, and those with PL-HM decreased from 22 to 5. However, the number of patients with no perception of light (NPL) increased from 8 to 14 at follow-up. The presenting VA and final VA were positively correlated (Spearman’s *r* = 0.53, p = < 0.001, CI [0.32, 0.69]).

**Table 2:** Association between VA at presentation and final visual outcome at 3 months

VA at presentation		VA at 3 months					
VA Category	Number of eyes	NPL Freq (%)	PL – HM Freq (%)	CF to < 6/60 Freq (%)	6/60 to < 6/12 Freq (%)	> 6/12 Freq (%)	P value
> 6/12	18	0	0	0	0	18(100)	Value = x^2^= 61.33P < 0.001
< 6/12 – 6/60	14	1 (7.1)	0	0	3 (21.4)	10(71.4)	
< 6/60 – CF	7	1 (14.3)	0	2(28.6)	0	4(57.1)	
PL/HM	22	5 (22.7)	5(22.7)	2(9.1)	3 (13.6)	7(31.8)	
NPL	8	7 (87.5)	0	0	0	1(12.5)	
**Total**	**69**	**14(20.3)**	**5(7.2%)**	**4(5.8)**	**6(8.7)**	**40(58.0)**	

Key: OTS- Ocular trauma score, NPL: No perception of light, PL: Perception of Light, HM: Hand motion, CF- Counting fingers VA: Visual acuity, p value- Chi squared test.  Spearman’s r=0.53, p=<.001, CI [0.32, 0.69]).

Table 3 demonstrates an association between ocular trauma score (OTS) and actual final visual acuity, as indicated by the Chi-squared test (X^2^= 96.21, *p* < 0.001). Additionally, a strong positive correlation was observed between OTS score and final visual acuity at 3 months (Spearman’s *r* = 0.60, *p* < 0.001, 95% CI [0.42, 0.74].). Patients with higher OTS values were more likely to achieve better visual acuity 3 months after treatment.

**Table 3:** Spearman’s correlation between OTS and final VA at 3 months

OTS Category	VA at 3 months						*P* value
	NPLFreq (%)	PL - HMFreq (%)	CF to <6/60Freq(%)	6/60 to < 6/12Freq(%)	*>* 6/12Freq (%)	TotalFreq (%)	
1 (0-44)	9(100)	0	0	0	0	9(100)	Spearman’s *r* = 0.60, *p* < 0.001, 95% CI [0.42, 0.74].).
2 (45-65)	4(33.3)	5 (41.7)	1 (8.3)	1 (8.3)	1 (8.3)	12(100)	
3 (66-80)	1(5.3)	0	3(15.8)	4 (21.1)	11(57.9)	19(100)	
4 (81-91)	0	0	0	1(8.3)	11(91.7)	12(100)	
5 (92-100)	0	0	0	0	17(100)	17(100)	
**Total**	**14(20.3)**	**5 (7.2)**	**4(5.8)**	**6(8.7)**	**40(58.0)**	**69(100)**	

Key: OTS- Ocular trauma score, NPL: No perception of light, PL: Perception of Light, HM: Hand motion, CF- Counting fingers VA: Visual acuity, Chi squared test : X^2^ =96.21, *p* < 0.001

**Fig. 3:**
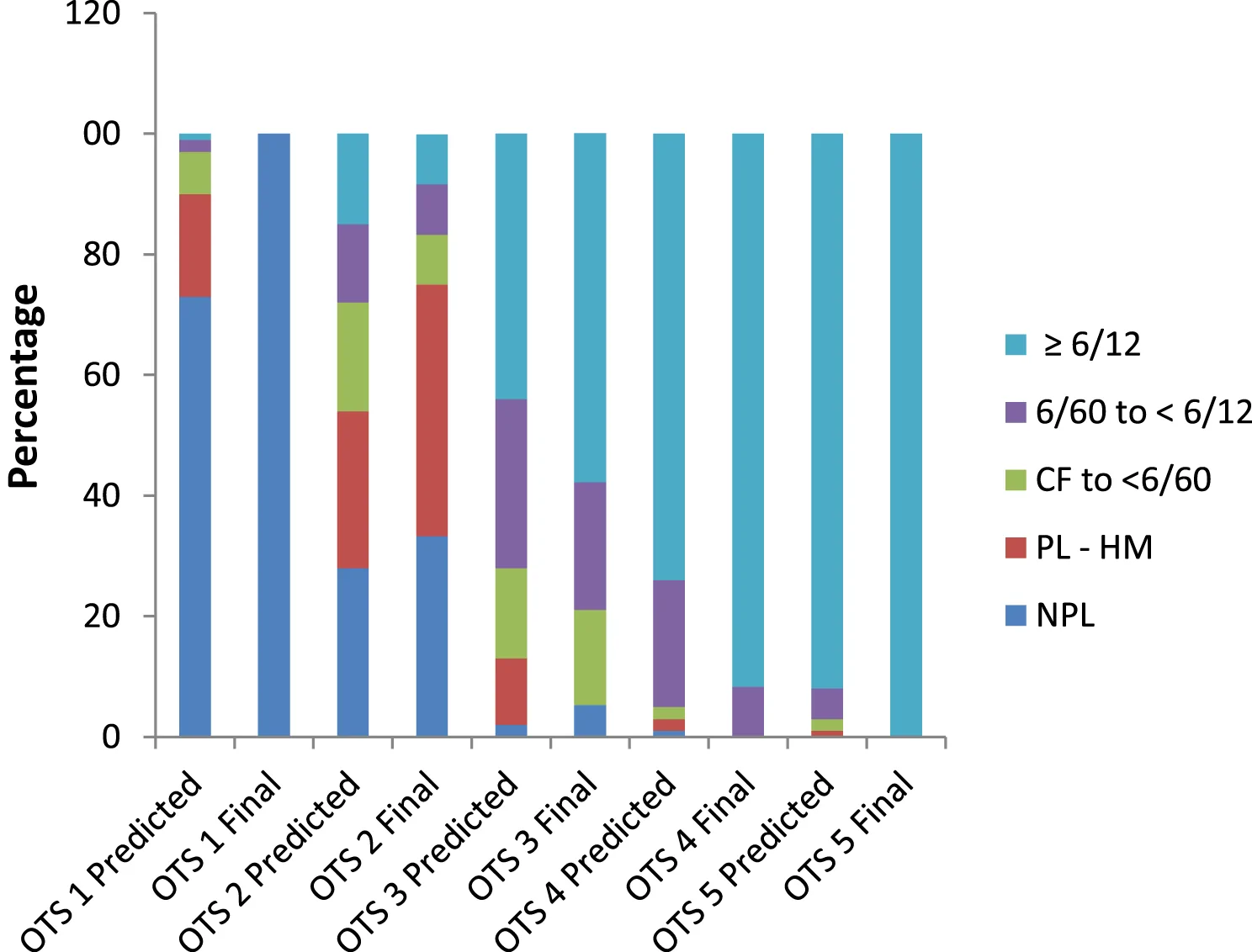
Category specific predicted versus final visual acuity accuracy

Figure 3 below shows OTS predicted visual acuity versus final visual acuity recorded 3 months after presentation for the 5 OTS categories.

Table 4 compares final visual acuity with OTS-predicted visual acuity. The ranked OTS-predicted mean visual acuity had a lower median value (OVAR; 4.01) than the ranked actual final mean visual acuity (AVAR; 4.26). The Mann-Whitney U-test indicated no statistically significant difference between OTS-predicted and actual final visual acuity at 3 months (*p* = 0.835) across all groups. Actual visual outcomes were marginally higher than predicted in Categories 3, 4, and 5, and slightly lower in Categories 1 and 2. However, differences between actual and predicted visual acuity were not statistically significant in any group. Overall, actual visual outcomes closely followed the predicted trend.

**Table 4:** Distribution of Actual final visual acuity compared to Kuhn’s OTS predicted visual acuity (in parentheses) for each OTS score

OTS Category	NPL		PL - HM		CF to < 6/60		6/60 to < 6/12		> 6/12		Total (n)	OVAR	AVAR	*p* value
	Final	OTS	Final	OTS	Final	OTS	Final	OTS	Final	OTS				
	(%)	(%)	(%)	(%)	(%)	(%)	(%)	(%)	(%)	(%)				
1	100	73	0	17	0	7	0	2	0	1	9 (9)	1.41	1	0.1
2	33.3	28	41.7	26	8.3	18	8.3	13	8.3	15	12(26)	2.61	2.17	0.131
3	5.3	2	0	11	15.8	15	21.1	28	57.9	44	19(81)	4.01	4.26	0.289
4	0	1	0	2	0	2	8.3	21	91.7	74	12(59)	4.65	4.92	0.401
5	0	0	0	1	0	2	0	5	100	92	17(85)	4.88	5	0.1

Key: OTS- Ocular trauma score , NPL: No perception of light, PL: Perception of Light, HM: Hand motion, CF- Counting fingers VA: Visual acuity ;OVAR- OTS predicted visual acuity rank, AVAR- mean actual final visual acuity rank; p value; Mann-Whitney U-Test performed for each OTS category

Approximately 38% of patients developed complications. Table 5 lists the complications observed at the 3-month follow-up. The most common complications were evisceration 5(16.7%), phthisis bulbi 5(16.7%), cataract 4 (13.3%), corneal scar 3 (10%), traumatic mydriasis 3 (10%), and retinal detachment 3 (10%). Of the eleven patients that presented with ruptured globe, the final vision was NPL in 9 eyes and PL/HM in two eyes. For five patients presenting with endophthalmitis, their final vision were NPL and PL/HM in four and one patient respectively. A patient who presented with CF to < 6/60 vision following a knife injury (corneo-scleral laceration, uveal prolapse, retinal detachment) also returned NPL three months later.

**Table 5:** Complications recorded at 3 months follow up

Complications	Frequency	Percentage (%)
Aphakia	1	3.3
Cataract	4	13.3
Corneal opacity	2	6.7
Corneal scar	3	10
Eviscerated globe	5	16.7
Macular hole	1	3.3
Macular scar	1	3.3
Optic atrophy	1	3.3
Posterior capsular rent	1	3.3
Phthisis bulbi	5	16.7
Retinal detachment	3	10
Traumatic mydriasis	3	10
**Total**	**30**	**100**

## Discussion

In this research we compared the OTS-predicted visual outcome with actual visual acuity three months after initial presentation at the hospital. Comparison was performed for both closed and open-globe injuries. To enable direct comparison, ranked actual and ranked predicted outcomes were calculated and categorized. Our study showed an association between presenting and final visual acuity and a correlation between the OTS scores and final visual outcome. The difference between OTS-predicted and actual final visual outcome was not significant statistically.

More than half (57.7%) of the patients presented with visual acuity of 6/60 or worse, underscoring the severity of vision loss following ocular injury and the need for prompt, effective treatment. In this study, 31.6% of patients presented within 24 h, and for majority (68.4%) of the patients, the emergency unit of the study hospital was their first point ​of presentation. This differs from findings by Khan et al. [18] in rural Bangladesh, where higher rates of self-treatment (45.5%), alternative care (42.1%) and unlicensed practitioner consultations (28.8%) were observed. Ajayi et al. [6]. in Nigeria, also reported that 69.4% of their participants used various medications before hospital presentation. Prompt specialist intervention in this setting may have contributed to favorable visual outcomes, as supported by some studies. For instance, in India [19], late presentation (more than 6 h post-injury) was associated with a 47% increased risk of blindness, although some studies [20, 21] documented significant visual improvement regardless of presentation timing. The results jointly emphasize substantial differences in care-seeking behaviors and the complex, multifactorial determinants of visual outcomes.

The majority of patients in this study demonstrated improved final visual acuity. At three-month follow-up, over half (58%) achieved a final visual acuity (VA) of 6/12 or better, while approximately one-third (33.3%) had a final VA of 6/60 or worse. These results are consistent with findings from similar studies [22, 23]. For example, Omolase et al. in Owo [22] reported that 50.8% of patients had a presenting VA of 6/18 or better, increasing to 77.3% after treatment. Additionally, VA less than 6/60 occurred in 37.1% at presentation, as compared to only 18.2% after treatment. Cillino et al. [23] in Italy observed that 48.3% achieved a final VA of 6/12 or better, and 15.5% had a final VA of less than 6/60. Grieshaber et al. [24] in South Africa found that, although 67.4% of injured children’s eyes had a VA less than 6/60 at presentation, after treatment, most (51%) achieved a final VA of 6/12 or better. These data indicate that appropriate intervention can restore good vision following ocular trauma. In contrast, Monsudi et al. [25] reported that 12.4% had a VA of 6/12 or better at presentation, and after follow-up, only 14% achieved a final VA of 6/12 or better, with 50% having a VA less than 6/60. Unlike the present study, which included both open- and closed-globe injuries, Monsudi et al. [25] focused exclusively on penetrating injuries, which may explain the poorer outcomes. Furthermore, open-globe injuries are generally associated with worse visual outcomes compared to closed-globe injuries [26].

A strong correlation was observed between OTS and visual outcome ( *r*= 0.60, *p* < 0.001). Patients presenting with a VA of 6/12 or better comprised 23.1% of the cohort. OTS categories 4 and 5 were identified in 39% of patients, and 58% achieved a favorable final VA. These outcomes are consistent with previous studies. Noble et al., Iwarati et al., and Meng et al. [21, 27, 28] also reported similar correlations. Patients with higher OTS values experienced better outcomes following both short-term (three months) and long-term (six months) treatment [29].

Perception of light/hand motion (PL-HM) was the most common presenting visual acuity (37.2%). The proportion of patients with poor visual acuity at presentation was lower than that reported by Mascaro et al. [17], where PL/HM accounted for 59%. The higher rate of poor visual acuity in their study was likely attributable to the exclusive inclusion of open-globe injuries. OTS category 3 (31.2%) was the most prevalent in this cohort. Similarly, Kuhn et al. [11] found that 38% of their patients were classified as OTS 3. The results indicate that both closed- and open-globe injuries could equally contribute to visual impairment and OTS classification.

The trend in visual outcomes predicted by OTS closely matched the results observed in this study. OTS predictions were slightly overestimated for groups 1 and 2 and underestimated for groups 3, 4, and 5; however, these discrepancies among predicted and actual outcomes were not statistically significant. Previous studies have reported varying predictive validity: Shretha et al. [30] found that predicted outcomes were achieved in 60% of cases; Munir et al. [31] observed variable accuracy for groups 1–3, and accurate predictions for groups 4 and 5; Chaudhary et al., Deud et al. and Bhat et al., [29, 32, 33] found no significant difference between predicted and final outcomes; and Noble et al. and Chaudhary et al. [21, 29] reported accurate prediction of visual outcomes for both open- and closed-globe injuries.

Visual outcomes at three months were comparable to those usually observed at six months. In this study, outcomes for OTS categories 3, 4, and 5 exceeded the predicted rates at six months. Specifically, final visual acuity (VA) of 6/12 or better was achieved in 57.9%, 91.7%, and 100% of patients in OTS categories 3, 4, and 5, respectively, compared with predicted rates of 44%, 74%, and 92%, respectively. These outcomes are supported by Chaudhary et al. [29] and Shrestha et al. [30], who reported that OTS could predict visual outcomes three months after injury. The use of OTS predictions at three months post-injury may provide a conservative estimate of visual outcomes.

The major complications in this study were evisceration, phthisis bulbi, and cataract. Cataracts were effectively managed with surgical intervention. Some other complications like endophthalmitis and retinal detachment resulted in permanent monocular visual loss. These complications explain why patients with final visual outcome of NPL increased from 8 (11.5%) to 14 (20.3%) three months later.

A primary limitation of this study was patient loss to follow-up, largely attributable to cultural beliefs and financial constraints. Patients that noticed improvement in vision may have felt no need for repeat hospital visits. Telephone reminders did not consistently ensure patient return for follow-up visits. The small sample size could have introduced statistical bias, potentially leading to overstated findings. Larger, multicenter or population-based studies in a similar resource-limited setting which may produce comparable results, is recommended.

## Conclusion

Mechanical ocular trauma was more prevalent among males and predominantly involved closed-globe injuries. The majority of injuries occurred in zones 1 and 11. A significant association was observed between presenting and final visual acuity at three months follow-up. A strong correlation was also identified between the ocular trauma score (OTS) and three-month visual outcomes. The trend in visual outcomes predicted by OTS was consistent with the findings of the present study. The ocular trauma score is a valuable predictor of visual outcome three months after both closed- and open-globe injuries in Nigerian patients. OTS can be applied to estimate the visual prognosis for mechanical injuries during the initial evaluation of trauma cases in the emergency department [34].

## Supplementary Information

Below is the link to the electronic supplementary material.

Supplementary Material 1

## Data Availability

The datasets used for the index study are available from the corresponding author upon reasonable request.

## Abbreviations

OTS: Ocular trauma score
BETTS: Birmingham eye trauma terminology system
VA: Visual acuity
NECK: National eye centre, kaduna
IOP: Intraocular pressure
CF: Counting fingers
HM: Hand motion
PL: Perception of light
NPL: No perception of light
APD: Afferent pupillary defect
IOFB: Intraocular foreign body
RD: Retinal detachment
OVAR: OTS predicted visual acuity rank
AVAR: Mean actual final visual acuity rank
