# Supplementary material for: Validation of ocular trauma score as a prognosticator of visual outcomes in open and closed-globe injuries at Kaduna, Nigeria
Source: BMC Ophthalmol. 2026 Nov 4;26:730. doi: 10.1186/s12886-026-05509-x (PMC13737647; doi:10.1186/s12886-026-05509-x)
Supplement: Supplementary file 1 [file 12886_2026_5509_MOESM1_ESM.docx]

**RESEARCH PROFORMA**

Topic: “Pattern and Visual Outcomes of Ocular Trauma at National Eye Centre Kaduna”.

**BIO DATA/HISTORY**

ID No:................................ Age..............................Gender: Male [ ] Female [ ]

Residential Address:.....................................................

TeI No:.....................................................

Occupation…………………………….Highest education attained …………………..

Date of Injury:..................................... Date of Presentation:....................................

Presenting Complaint(s)…........................................................................................

Injured eye:	RE [ ]	LE [ ]	BE [ ]

Place of Injury (Home/School/Road/Sports/Work etc):……........................................

Any use of eye protective goggles?............................................................................

Object of injury…………….……………………………………………………………

Mechanism/Cause of injury: ……..............................................................................

Past History of Ocular Injury......................................................................................

Any Pre-existing eye disease?.....................................................................................

**Visual acuity at presentation.**RE [ ]	LE [ ]

**Type of Injury:**

Open Globe	Closed Globe

Laceration	Lamellar Laceration

Rupture	Contusion

IOFB

Penetrating

Perforation

**Zone of Injury**

Zone	1

Zone 11

Zone 111

**Ocular examination at presentation	Ocular examination at 3 months**

| Visual Acuity | Unaided |  |
| --- | --- | --- |
|  | Pin Hole |  |
| Globe Rupture |  | |
| Endophthalmitis |  | |
| Perforating Injury |  | |
| Retinal Detachment |  | |
| RAPD |  | |
| Others (Specify) |  | |
| **OTS** |  | |

| Visual Acuity | Unaided |  |
| --- | --- | --- |
|  | Best Corrected |  |
| Treatment Received | Conservative |  |
|  | Surgical |  |
| Complication |  | |

**PATIENT INFORMATION /CONSENT SHEET**

Topic: “Pattern and Visual Outcomes of Ocular Trauma at National Eye Centre Kaduna”.

**INTRODUCTION**

My name is Dr Ahmed Saidu. I am carrying out above titled research as part of my dissertation in part fulfilment for the award of fellowship of the National Postgraduate Medical College of Nigeria in ophthalmology.

**Background**

Ocular trauma is one of the major causes of monocular visual impairment and blindness worldwide. Ocular trauma is brought about by a variety of causes, ranging from a trivial domestic accident to a more serious industrial or road traffic accident. The outcome of ocular trauma is dependent on many factors such as the mechanisms/causes of the injury; time elapsed from the time of injury to the time of presentation and also the quality of care given to the patient. Therefore, the aim of this study is to provide data on the pattern and outcome of ocular trauma at the National Eye Centre Kaduna, through a prospective study. This will help to improve the treatment of ocular trauma and also prevent occurrences of ocular injuries among the population and hence reduce the incidences of blindness due to ocular injury in the future.

**PARTICIPATION**

Participation is purely voluntary and is indicated by signing the informed consent. If enrolled, you will be fully registered with a study ID number and your telephone number will be documented for follow-up purposes. Questionnaires will be administered to you followed by a complete eye examination including some that may cause some degree of discomfort. None of the eye examinations is harmful. All information collected will be treated with uttermost confidentiality and results obtained will be used to improve eye care. Your refusal to participate will not affect your right to treatment in the hospital.

**FINANCIAL ARRANGEMENT**

The research is for academic purpose only, with no financial attachment. You will not pay for the various examinations.

**CONSENT TO PARTICIPATE IN RESEARCH**

I......................................................................................................................, after being fully briefed of the research titled “Pattern and Visual Outcomes of Ocular Trauma at National Eye Centre, Kaduna” have agreed to volunteer myself to participate fully for that purpose on the understanding that I shall be entitled to opt out of the research or withdraw my consent at any time. I understand that the study involves history taking, questionnaires, eye examination and other necessary investigations, the nature of which has been filly explained to me. I also acknowledge that the risks and benefits have been explained to me. I am also aware that the information obtained during this study will be treated with utmost confidentiality and that the outcome of this research will be published and help to improve eye care. In the event that I opt out, I understand it will not affect the care I will receive in this hospital or elsewhere.

Signature/thumbprint___________________

Date:______________________
